# Microscopic Analysis of Severe Structural Rearrangements of the Plant Endoplasmic Reticulum and Golgi Caused by Overexpression of *Poa semilatent virus* Movement Protein

**DOI:** 10.1100/2012/416076

**Published:** 2012-01-04

**Authors:** Andrey G. Solovyev, Joachim Schiemann, Sergey Y. Morozov

**Affiliations:** ^1^A. N. Belozersky Institute of Physico-Chemical Biology, Moscow State University, 119992 Moscow, Russia; ^2^Institute of Agricultural Biotechnology, Russian Academy of Agricultural Sciences, Timiryazevskaya 42, 127550 Moscow, Russia; ^3^Julius Kühn Institute (JKI), Federal Research Centre for Cultivated Plants, Institute for Biosafety of Genetically Modified Plants, Erwin-Baur-Street 27, 06484 Quedlinburg, Germany; ^4^Department of Virology, Biological Faculty, Moscow State University, 119992 Moscow, Russia

## Abstract

Cell-to-cell transport of plant viruses is mediated by virus-encoded movement proteins and occurs through plasmodesmata interconnecting neighboring cells in plant tissues. Three movement proteins coded by the “triple gene block” (TGB) and named TGBp1, TGBp2 and TGBp3 have distinct functions in viral transport. TGBp1 binds viral genomic RNAs to form ribonucleoprotein complexes representing the transport form of viral genome, while TGBp2 and TGBp3 are necessary for intracellular delivery of such complexes to plasmodesmata. Recently, it was revealed that overexpression of *Potato virus X* TGBp3 triggers the unfolded protein response mitigating the endoplasmic reticulum (ER) stress leading to cell death if this protein reaches high levels in the ER. Here we report microscopic studies of the influence of the *Poa semilatent hordeivirus* TGBp3 overexpressed in *Nicotiana benthamiana* epidermal cells by particle bombardment on cell endomembranes and demonstrate that the protein C-terminal transmembrane segment contains a determinant responsible for vesiculation and coalescence of the endoplasmic reticulum and Golgi presumably accompanying the ER stress that can be induced upon high-level TGBp3 expression.

## 1. Introduction

Transport of plant virus genomes from infected to neighboring healthy cells, termed “virus cell-to-cell movement”, occurs through plasmodesmata and involves dedicated virus-encoded movement proteins (MPs) [1, 2]. Many positive-stranded RNA phytoviruses possess three MPs encoded by overlapping genes organized in a “triple-gene block” (TGB) [3, 4]. In recent years, the molecular mechanism of TGB-mediated cell-to-cell movement was studied for several viral genera including the genera Hordeivirus and Potexvirus [4, 5]. 

The hordeiviral TGB proteins, termed TGBp1, TGBp2, and TGBp3, are extensively characterized both structurally and functionally [5]. TGBp1, the largest of the TGB proteins with the molecular mass of 50 to 63 kDa in different hordeiviruses, binds viral genomic RNAs to form ribonucleoprotein complexes (RNPs), which are believed to be a transport form of the viral genome [5]. TGBp2 and TGBp3 are smaller (14–18 kDa) proteins integrated into cell membranes due to two hydrophobic segments found in each of these proteins [4, 5]. Subcellular localization studies employing fusions of TGBp2 and TGBp3 to fluorescent reporter proteins revealed that (i) TGBp2 alone is localized to the endoplasmic reticulum (ER) structures and ER-associated vesicles; (ii) TGBp3 is localized to cell wall-appressed peripheral membrane bodies (PMBs) located in close vicinity of plasmodesmata and containing an ER marker that points to their ER origin; (iii) in the presence of TGBp3, TGBp2 is also targeted to PMBs [4–6]. Furthermore, BSMV TGBp1, which is localized to cytoplasmic bodies of unknown nature when expressed alone, is targeted to plasmodesmata in the presence of both TGBp2 and TGBp3 [7]. Additionally, as it is demonstrated for *Potato mop-top virus*, TGBp2 and TGBp3 not only direct TGBp1 to plasmodesmata but also mediate its transport through plasmodesmata to neighboring cells [8, 9]. Analysis of the pathway of TGBp3 intracellular transport from sites of its cotranslational integration into the ER membrane to plasmodesmata-associated sites reveals that it does not involve exit from the ER in COPII-coated transport vesicles and thus employs an unconventional mechanism [10], which can involve a lateral diffusion of protein molecules in the lipid bilayer of ER membranes as it is proposed for the intracellular transport of the MP of *Tobacco mosaic virus* [11]. Deletion analysis of hordeivirus TGBp3 reveals that the signal of plasmodesmata targeting is composite and consists of at least two parts, the central hydrophilic region containing an invariant pentapeptide YQDLN and the C-terminal transmembrane domain [10]. Recently, we have demonstrated that the YQDLN-containing conserved region is essential for TGBp3 incorporation into high-molecular-mass protein complexes representing the form in which TGBp3 is found in virus-infected plants [12]. Most importantly, the formation of such complexes is necessary for entering the TGBp3-specific pathway of intracellular transport and protein delivery to PMBs. On the other hand, the C-terminal transmembrane segment is a *bona fide* signal of TGBp3 intracellular transport since the transport to PMBs of the protein with disabled YQDLN-containing region is restored by fusion to a heterologous peptide capable of multimer formation [12].

 The ratio for accumulation of TGBp1, TGBp2, and TGBp3 proteins in infected plant tissues is estimated to be 100 : 10 : 1, respectively [5]. Increase of the TGBp3 to TGBp2 ratio leading to over-expression of TGB3 has been shown to interfere with protein plasmodesmata targeting and virus cell-to-cell movement [4]. Recently, *Potato virus X* (PVX) TGBp3 was reported to stimulate unfolded protein response (UPR) when expressed from the heterologous virus vectors [13]. Upon protein overexpression, the ER protein folding machinery reaches a limit, as the demands for protein folding exceed the capacity of the system. Under these conditions, misfolded or unfolded proteins accumulate in the ER, triggering UPR [14]. UPR mitigates the ER stress by upregulating the expression of genes encoding components of the protein folding machinery or the ER-associated degradation system.

 Despite recent progress in our understanding of virus protein- and stress-induced plant UPR at biochemical level, little is known about the influence of these stress conditions on the structure and morphology of cell endomembrane system. In this paper we report the effect of wild type (nonfused) TGBp3 of *Poa semilatent virus* (PSLV, genus Hordeivirus) on the ER and Golgi in cells transiently expressing this protein after particle bombardment with a 35S-promoter-driven expression vector.

## 2. Materials and Methods

### 2.1. Particle Bombardment

Wild-type PSLV TGBp3 and its mutant, as well as marker proteins, were expressed in epidermal cells of *Nicotiana benthamiana *leaves by particle bombardment with recombinant plasmids performed using the flying disc method with a high-pressure helium-based PDS-1000 system (Bio-Rad) as described in [17].

### 2.2. Plasmid Constructs

Recombinant plasmids pRT-GFP-18K encoding a GFP fusion of the PSLV TGBp3, pRT-18K encoding the nonfused PSLV TGBp3, pRT-GFP-18Kmut62 encoding a GFP-fused TGBp3 mutant [6], pRT-m-GFP5-ER encoding an ER marker [15], and pRT-ST-YFP encoding a Golgi marker [16] have been described earlier. To obtain the mutant 18KIId8 the TGBp3 gene was amplified with plus-sense primer Left [6] and a minus-sense primer 5′-GCTCTAGATTACTTGAATAATAAACCTACATAAAACTTAAGAG. *Bam*HI/*Xba*I-digested product was cloned into similarly digested pRT-GFP-18K to replace the wild type sequence. To generate YFP fusions of TGBp3 derivatives, the GFP gene was replaced with the YFP gene using appropriate restriction sites.

### 2.3. Cell Imaging

Imaging of bombarded cells was carried out with a Leica TCS SP2 system as described in [18]. GFP was visualized with an argon ion laser at 488 nm and an acquisition window of 500–530 nm. YFP was visualized with an argon ion laser at 514 nm and an acquisition window of 525–575 nm. For imaging of coexpressed yellow fluorescent protein (YFP) and GFP constructs, argon ion laser-excitation lines (488 nm for GFP and 514 nm for YFP) were used alternately. Accordingly, the fluorescence of GFP and YFP was detected alternately by using the “switching between lines” option of the confocal system in the 496–510 nm acquisition window for GFP and the 560–615 nm window for YFP. The software package provided by the manufacturer was used for projections of serial optical sections and image processing.

## 3. Results

### 3.1. Reorganization of the ER and Golgi Structures in the Presence of the Wild Type PSLV TGBp3

**Figure 1:**
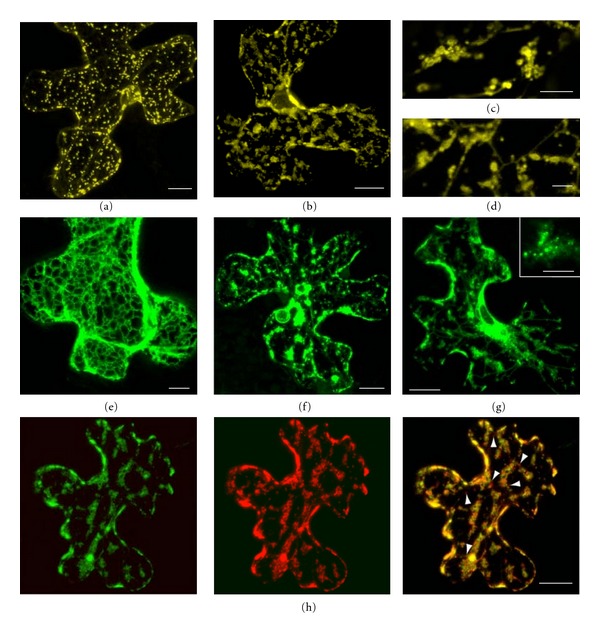
Co-expression of non-fused 18 K with the ER and Golgi markers in bombarded epidermal cells of *N. benthamiana* leaves. (a) ST-YFP. (b)–(d) ST-YFP + 18 K. (e) m-GFP5-ER. (f) and (g) m-GFP5-ER + 18 K. (h) ST-YFP + m-GFP5-ER + 18 K. In (h), GFP signal is shown in the left panel, YFP signal—in the middle panels, and the merged image—in the right panel. All images except (b) and (c) and the insert in (g) are reconstructed by superposition of series of confocal optical sections. Arrowheads in (h) point to round structures of 0.5–1.0 *μ*m in diameter presumably representing Golgi stacks remained unaffected upon the 18 K expression. Scale bar: 20 *μ*m in (a), (b), (f), (g), and (h); 10 *μ*m in (e); 4 *μ*m in (c) and (d); 3 *μ*m in the insert in (g).

During virus infection, hordeivirus TGBp3 is expressed at very low levels [5], and immunological detection of TGBp3 is only possible in samples highly enriched in cell membranes [12]. Therefore, for studies of the TGBp3 subcellular localization we have employed GFP-fused TGBp3 expressed in plants in the absence of viral infection [6, 10, 15, 19]. In particle bombardment experiments, we have found that the GFP-fused PSLV TGBp3 (18 K) was colocalized with an ER marker in PMBs [15]. In this paper we analyzed the effect of the nonfused 18 K expressed by particle bombardment in the absence of other viral products on the morphology of the ER and Golgi. 18 K was coexpressed with the ER and Golgi marker proteins in epidermal cells of *N. benthamiana* leaves by particle bombardment with 35S promoter-driven expression vectors. This method ensures co-expression of two proteins in all transfected cells [6, 9, 15]. Co-expression of 18 K with ST-YFP revealed considerable changes in the localization of this Golgi marker. Instead of numerous motile Golgi structures of regular spherical shape visible in control cells expressing only ST-YFP (Figure 1(a)), ST-YFP co-expressed with the non-fused 18 K was found in immobile groups of irregularly shaped vesicular structures of different sizes forming large “islands” sometimes interconnected by rare ST-YFP-containing membrane tubules resembling the tubules of cortical ER (Figures 1(b)–1(d)). Additionally, ST-YFP accumulated in the nuclear envelope (Figure 1(b)). In cells coexpressing the nonfused 18 K with the ER marker m-GFP5-ER the typical polygonal network of cortical ER (Figure 1(e)) was not observed. Instead, the fluorescent marker was localized in groups of granular structures, in the nuclear envelope, and in rare residual ER tubules interconnecting the granular clusters (Figures 1(f) and 1(g)). It should be emphasized that our previous studies revealed that 18 K N-terminally fused to fluorescent proteins GFP and DsRed did not exhibit any effect on the general endomembrane system structure in similar experimental conditions [10, 15]. Therefore, one can propose that the mode of 18 K interaction with membranes can be altered when the protein N-terminus is fused to a reporter protein.

 Since ST-YFP and m-GFP5-ER were localized in similar structures upon their individual coexpression with 18 K, we further analyzed weather the ER and Golgi markers are colocalized upon co-expression with 18 K. Independent detection of GFP and YFP signals and subsequent image superposition revealed the colocalization of GFP and YFP in the vesicular clusters (Figure 1(h)). Additionally, the YFP signal (but not the GFP signal) was found in some round structures of 0.5–1.0 *μ*m in diameter (Figure 1(h)), which presumably represented Golgi stacks remained unaffected upon the 18 K expression.

 Collectively, these data indicate that the nonfused 18 K protein expressed in plant cells by particle bombardment with a 35S-promoter-driven vector in the absence of other viral proteins can induce reorganization of the cortical ER and Golgi structures and their coalescence resulting in formation of the vesicular clusters. We hypothesize that TGBp3 primarily affects the ER that results in its vesiculation and presumably in a blockage of ER-to-Golgi transport that would lead to disintegration of Golgi stacks and accumulation of Golgi-specific proteins in the ER-derived vesicular clusters [20, 21].

### 3.2. A Mutation in the C-Terminal Transmembrane Domain Influences TGBp3-Induced Reorganization of the Cell Endomembrane System

**Figure 2:**
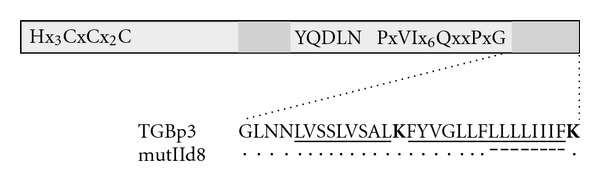
Schematic representation of TGBp3 mutant 18KmutIId8. The box represents TGBp3 sequence. Hydrophobic sequence segments are shown as dark grey boxes. Conserved amino acid motifs are indicated. Sequences of the C-terminal hydrophobic segment in the wild-type protein and 18KmutIId8 are shown below the protein scheme. Hydrophobic regions are underlined; the positively charged Lys residues are shown in bold. Dots show identical residues, and dashes indicate deletions.

**Figure 3:**
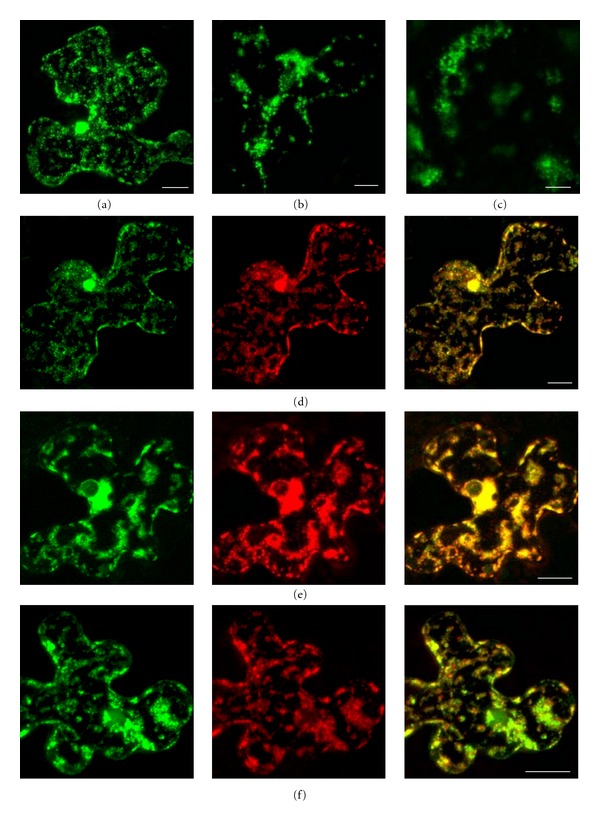
Co-expression of 18KIId8 and its fusions with the ER and Golgi markers in bombarded epidermal cells of *N. benthamiana* leaves. (a)–(c) GFP-18KIId8. (d) ST-YFP + GFP-18KIId8. (e) m-GFP5-ER + YFP-18KIId8. (f) m-GFP5-ER + ST-YFP + 18KIId8. In (d)–(f), GFP signal is shown in the left panels, YFP signal—in the middle panels, and merged images—in the right panels. All images except (c) are reconstructed by superposition of series of confocal optical sections. (c) represents a single optical section in a cell peripheral region. Scale bar: 20 *μ*m in (a), (d)–(f); 10 *μ*m in (b); 4 *μ*m in (c).

Previously we reported that a deletion of four C-terminal amino acid residues of the second transmembrane domain blocked the GFP-18 K ability for transport to cell peripheral compartments [10]. Here we analyzed a mutant with a longer deletion in this transmembrane segment, 18KIId8 with eight residues deleted (Figure 2). GFP-18KIId8 was not associated with PMBs and localized in numerous granular structures often grouped in clusters (Figures 3(a)–3(c)), that resembled localization of m-GFP5-ER and ST-YFP in the presence of non-fused 18 K (Figure 1). We therefore analyzed whether 18KIId8 fused to a fluorescent reporter can affect the ER and Golgi similarly to the nonfused wild-type protein. Co-expression of YFP-18KIId8 with m-GFP5-ER revealed that the GFP and YFP signals were colocalized in granular clusters in the cytoplasm (Figure 3(e)). Similar colocalization was revealed for co-expression of ST-YFP and GFP-18KIId8 (Figure 3(d)). To determine whether non-fused 18KIId8 can induce the coalescence of structures derived from the ER and Golgi, 18KIId8 was coexpressed with both m-GFP5-ER and ST-YFP. It was found that GFP and YFP signals in such cells mostly overlapped (Figure 3(f)). One can conclude that GFP-fused 18KIId8 with the deletion of eight residues in the C-terminal transmembrane segment, in contrast to GFP-18K, retains the ability of wild-type 18 K to induce, upon high-level expression, morphological changes in the ER and Golgi structures. Presumably, the 18 K C-terminal transmembrane domain represents an important determinant involved in the interaction with ER and responsible for the observed effect of 18 K high-level expression on the ER and Golgi.

## 4. Discussion

The data presented in this paper show dramatic effects of the wild-type 18 K and GFP-18KIId8 on the cell endomembranes. It should be emphasized that the TGBp3 expression levels reached in bombardment experiments most likely considerably exceed those in virus infection. Therefore, the coalescence of the ER and Golgi structures could be considered as a result of an overexpression of TGBp3. Such severe influence on the cell endomembranes could account for the blockage of plasmodesmata targeting and cell-to-cell movement previously observed upon increase of TGBp3 to TGBp2 ratio in beny-, pomo-, and hordeiviruses [4, 5]. We hypothesize that the observed effects of 18 K and GFP-18KIId8 recapitulate, though in a hypertrophic way, the processes of UPR and cell death-causing ER-stress that take place in virus-infected cells [13]. As a result of high-level expression in bombarded cells, demands for protein folding can exceed the functional capability of protein folding machinery, resulting in the ER stress and leading to severe structural perturbations in the plant endoplasmic reticulum and Golgi [13, 22]. It was found in animal and yeast models that protein over-expression affected global ER and Golgi structure and resulted in the biogenesis of novel membrane arrays with Golgi and ER hybrid composition. In fact, a number of ER and Golgi resident proteins together with itinerant proteins that normally cycle between ER and Golgi were localized in the proliferated stacked membranes under the ER stress conditions [22, 23].

 An alternative mechanism for the TGBp3 influence on the cell endomembrane system and, in particular, the mechanism of vesiculation of the ER structures can be proposed on the basis of the recent finding that TGBp3 encoded by *Bamboo mosaic virus* is colocalized in PMBs with cell proteins called Rtn1 and Yop1 [24]. The reticulon (Rtn1) and DP1/Yop1 belong to two families of integral ER membrane proteins that facilitate formation of highly curved membrane tubules and thus take a part in shaping the cortical ER [25, 26]. There are two features shared by (Rtn1) and DP1/Yop1 on one hand and hordeivirus TGBp3 on another. First, similarly to the hordeivirus TGBp3, the “reticulon homology domain” shared by Rtn1 and DP1/Yop1 comprises two transmembrane segments separated by a conserved hydrophilic region and can therefore possess a TGBp3-like topology in the ER membrane. Second, similarly to Rtn1 and DP1/Yop1, TGBp3 can form high-molecular weight complexes in cell membranes [8]. It could be hypothesized that the mechanism of TGBp3 targeting to PMBs involves sorting to highly curved membrane compartments where Rtn1 and DP1/Yop1 reside. On the other hand, one can presume that, similarly to reticulons, TGBp3 expressed to high levels can itself generate a membrane curvature that would result in general changes in the ER morphology. Similarly, the expression of the *Arabidopsis thaliana* reticulon RTNLB13 in plant cells results in vesiculation of the cortical ER tubules, and the extent of vesiculation correlates with the level of RTNLB13 expression [27]. Moreover, in striking similarity to TGBp3, the RTNLB13-induced vesiculation was significantly milder when RTNLB13 was fused to a fluorescent protein [27].
